# Are There Too Many Dentists?

**Published:** 1887-11

**Authors:** 


ARTICLE IX.

### ARE THERE TOO MANY DENTISTS?

This question is supposed to have been propounded originally by a dentist. Whoever he was, it is probable that his own answer to the question could be found by transposing the first two words of the query. Figures do not always express truth, but they are of some value. Divide the population of the United States in 1880 (50,000,-000) by the whole number of dentists (15,000), and we have 3,333 as the proportion of patients to each dentist. Deduct from this number the paupers, the insane, the infants, and the average would probably be reduced at least one-half. If we consider further, the large proportion of visitants to dental offices who occupy time and attention without affording the dentist opportunity for a fee, another reduction from the average must be made. So much for the discouraging features of this question. Other statistics might be furnished of a more encouraging character. It seems that a multitude of dentists create a demand, instead of simply meeting it. This is more particularly the case than in the practice of medicine. Communities manage to live without dentists, which ought to support half a dozen. Facts may be supplied to prove that quality and not numbers in a community most affects the dentist's prosperity. In the East it is said that, despite close competition in prices, dentists, as a class, are prosperous. This holds good in towns and villages. From all that might be gathered, it would appear that there are not too many dentists, but there are too many who do not appreciate dental services. The further west we go the less evidence of prosperity we see among dentists, excepting in the money centres. There are many exceptions, but we are speaking now of the general average. Dentistry not infrequently goes hand in hand with some light trade, or merchandising, in states this side of the Mississippi, too. In view of these facts it is not strange that dentists everywhere look with longing eyes toward the cities. They expect there to find a class of appreciative patrons. Encouraged by promises of aid and influence from friends among the physicians, they crowd in and take their chances. After a time, perhaps, they become aware that even in cities a small proportion, comparatively, of all the population seek the dentist's services. And that small proportion is already attached to the established dentists. The trans-Mississippi region is destined to furnish a fine field for the coming generations of dentists. The enormous influx of emigrants from abroad is, thus far, only the vanguard of the army which will cover the western plains during the next decade or two. From 1870 to 1880 Europe laid nearly 40,000 miles of railway, only 2,000 miles less than the United States constructed. This means increased facilities for poor emigrants to reach the sea-board. Steerage rates from Liverpool have been reduced to $8.00. A few years ago it was $35.00. It is estimated that with the opening of the 20th century we will have a foreign population of 43,000,000. Nine-tenths of these emigrants go west. They will cover the western plains, and hundreds of growing towns will assume the proportions of cities. The next twenty years, it is predicted, will constitute one of the most remarkable epochs in the world's history, as afforded by the spectacle of the unprecedented development of America's great West. These foreign elements will not in themselves, perhaps, directly afford encouragement to the coming dentists. But they will develop the country, and that will attract native emigration more than ever, to develop the cities and establish trade and manufacture. The question, Are there too many dentists, seems to be premature.—*Editorial in Cincinnati Medical and Dental Journal.*

